# Liquid Crystalline Nanoparticles as an Ophthalmic Delivery System for Tetrandrine: Development, Characterization, and In Vitro and In Vivo Evaluation

**DOI:** 10.1186/s11671-016-1471-0

**Published:** 2016-05-17

**Authors:** Rui Liu, Shuangshuang Wang, Shiming Fang, Jialu Wang, Jingjing Chen, Xingguo Huang, Xin He, Changxiao Liu

**Affiliations:** School of Chinese Materia Medica, Tianjin University of Traditional Chinese Medicine, 312 Anshanwest Road, Nankai District, Tianjin, 300193 China; Tianjin State Key Laboratory of Modern Chinese Medicine, 312 Anshanwest Road, Nankai District, Tianjin, 300193 China; State Key Laboratory of Drug Delivery Technology and Pharmacokinetics, Tianjin Institute of Pharmaceutical Research, 308 Anshanwest Road, Nankai District, Tianjin, 300193 China

**Keywords:** Liquid crystalline nanoparticles, Microdialysis, Pharmacokinetics, Bioavailability, Central composite design, Ophthalmic delivery, Pre-ocular retention, Corneal permeability, Glyceryl monoolein, Tetrandrine

## Abstract

The purpose of this study was to develop novel liquid crystalline nanoparticles (LCNPs) that display improved pre-ocular residence time and ocular bioavailability and that can be used as an ophthalmic delivery system for tetrandrine (TET). The delivery system consisted of three primary components, including glyceryl monoolein, poloxamer 407, and water, and two secondary components, including Gelucire 44/14 and amphipathic octadecyl-quaternized carboxymethyl chitosan. The amount of TET, the amount of glyceryl monoolein, and the ratio of poloxamer 407 to glyceryl monoolein were selected as the factors that were used to optimize the dependent variables, which included encapsulation efficiency and drug loading. A three-factor, five-level central composite design was constructed to optimize the formulation. TET-loaded LCNPs (TET-LCNPs) were characterized to determine their particle size, zeta potential, entrapment efficiency, drug loading capacity, particle morphology, inner crystalline structure, and in vitro drug release profile. Corneal permeation in excised rabbit corneas was evaluated. Pre-ocular retention was determined using a noninvasive fluorescence imaging system. Finally, pharmacokinetic study in the aqueous humor was performed by microdialysis technique. The optimal formulation had a mean particle size of 170.0 ± 13.34 nm, a homogeneous distribution with polydispersity index of 0.166 ± 0.02, a positive surface charge with a zeta potential of 29.3 ± 1.25 mV, a high entrapment efficiency of 95.46 ± 4.13 %, and a drug loading rate of 1.63 ± 0.07 %. Transmission electron microscopy showed spherical particles that had smooth surfaces. Small-angle X-ray scattering profiles revealed an inverted hexagonal phase. The in vitro release assays showed a sustained drug release profile. A corneal permeation study showed that the apparent permeability coefficient of the optimal formulation was 2.03-fold higher than that of the TET solution. Pre-ocular retention capacity study indicated that the retention of LCNPs was significantly longer than that of the solution (*p* < 0.01). In addition, a pharmacokinetic study of rabbit aqueous humors demonstrated that the TET-LCNPs showed 2.65-fold higher ocular bioavailability than that of TET solution. In conclusion, a LCNP system could be a promising method for increasing the ocular bioavailability of TET by enhancing its retention time and permeation into the cornea.

## Background

Among ocular drug delivery systems, the topical application of drug solutions in eye drops is the most common and accepted method of treating ocular diseases. However, solutions contained in drops typically display poor corneal penetration and have short pre-ocular retention times. To overcome these shortcomings, emulsions, liposomes, nanoparticles, microspheres, nanocapsules, and micelles are being developed as new ophthalmic delivery systems [1–6]. These systems can enhance ocular drug bioavailability by promoting transcorneal penetration, but as with traditional eye drops, the same rapid rates of drainage that result in the rapid pre-corneal loss of the drug and consequently low bioavailability have been observed in these formulations.

Liquid crystalline nanoparticles (LCNPs) are formed by some amphiphilic lipids in the presence of excess water, and they have therefore been proposed as potential drug delivery vehicles [7]. LCNPs offer higher encapsulation than other drug delivery systems because they have a higher proportion of lipids in the particle and therefore present a larger surface area. Moreover, previous research studies have revealed that LCNPs can incorporate large amounts of drugs with varying physicochemical properties. Taking into account the bioadhesive nature, nontoxicity, and sustained-release behavior, LCNPs show great promise as vehicles for the ocular administration of drugs. Furthermore, research of LCNPs as a drug delivery system has involved oral, percutaneous, and intravenous routes of administration [8–10].

The lipid that is most widely used to construct LCNP systems is glyceryl monooleate (GMO), which displays a biofilm-like microstructure that enables the lipid bilayer of GMO to fuse with epithelial cells. Furthermore, GMO, as a nontoxic, biodegradable, and biocompatible material, has demonstrated great potential for use in pharmaceutical applications. It spontaneously forms a well-ordered liquid crystal phase in water. According to differences in their internal structures, the liquid crystalline phases of these formulations include a lamellar phase (Lа), an inverted hexagonal phase (H_2_), and a cubic phase (V_2_) [11–13]. Among these phases, the non-lamellar phase is the most extensively studied phase in pharmaceutical research [14].

TET, (1β)-6,6′,7,12-tetramethoxy-2,2'-dimethylberbaman(S, S)-(+)-tetrandrine, is an alkaloid that is extracted from the Chinese medicinal herb *Radix Stephania tetrandra S.* that has been used clinically to treat chronic keratitis, cataracts, retinopathy, and glaucoma [15–17]. Unfortunately, although TET has many pharmacological effects, its therapeutic efficacy is greatly limited because of its low aqueous solubility.

Thus, it is desirable to design a novel ocular drug delivery system that not only increases the aqueous solubility of TET but also overcomes the shortcomings of conventional dosage forms. The purpose of this study was to design a novel ocular delivery system to improve the ocular bioavailability of TET. In this study, TET-loaded LCNPs were prepared and investigated to determine their physicochemical properties, including their entrapment efficiency, drug loading capacity, drug utilization, zeta potential, particle size, polydispersity index, particle morphology, and crystalline lattice structure. In vitro drug release from the LCNP system was determined using a dynamic dialysis method. An in vitro penetration study was performed using freshly excised rabbit corneas. Retention capacity was assessed using a noninvasive fluorescence imaging system. Finally, in vivo aqueous humor pharmacokinetics was investigated using a microdialysis method.

## Methods

### Materials

GMO was purchased from Adamas Reagent Co., Ltd. (Shanghai, China). Poloxamer 407 (F127) was obtained from BASF (Ludwigshafen, Germany). Tetrandrine was purchased from Jintai Biological Engineering Co., Ltd. (Shanxi, China). Gelucire 44/14 was a gift of Gattefosse S.A. (Saint-Priest, France). Amphipathic octadecyl-quaternized carboxymethyl chitosan (QACMC) was supplied by Nantong Lushen Biological Engineering Co., Ltd. (Jiangsu, China). All other chemicals and reagents were of analytical grade.

### Animals

New Zealand white rabbits weighing 2.5–3.0 kg were used in the present study. The animals were housed at 25 ± 1 °C in 50 ± 5 % relative humidity with access to food and water ad libitum. All animal experiments were reviewed and approved by the Animal Ethical Committee at the Tianjin University of Traditional Chinese Medicine.

### Central Composite Factorial Design

During the preliminary study, the effect of each parameter on the physicochemical properties of the TET-LCNPs was assessed. To determine the optimal conditions for the technical procedure, a total of 20 experimental runs were performed according to the central composite design principles using Design-Expert 8.06 (Stat Ease, Inc., Minneapolis, USA) as described in Table 1. A three-factor, five-level central composite design was generated to optimize the TET-LCNP preparation. The study design involved investigating the effects of three independent variables, including the amount of TET (*X*_1_), the amount of GMO (*X*_2_), and the F127/GMO ratio (*X*_3_). The dependent variables were the entrapment efficiency (*Y*_1_) and drug loading capacity (*Y*_2_) of the TET-LCNPs.

**Table 1:** Independent variables and levels of experiment design

Independent variables	Levels				
	−1.682	−1	0	1	1.682
*X*_1_: amount of TET (mg)	5	6.01	7.5	8.99	10
*X*_2_: amount of GMO (mg)	200	260.81	350	439.19	500
*X*_3_: F127/GMO (%)	9	10.22	12	13.78	15

A three-factor, five-level central composite design was generated to optimize the TET-LCNP preparation. The study design involved investigating the effects of three independent variables, including the amount of TET (*X*_1_), the amount of GMO (*X*_2_), and the F127/GMO ratio (*X*_3_). The dependent variables were the entrapment efficiency (*Y*_1_) and drug loading capacity (*Y*_2_) of the TET-LCNPs

### Preparation of TET-LCNPs

GMO, F127, and TET were dissolved in ethanol. The subsequent evaporation of the solvent left the oil phase. The QACMC and Gelucire 44/14 were dissolved in water to form the water phase. Thereafter, the water phase was dropped into the oil phase, and the mixture was roughly mixed for 2 min at 75 °C using a high-shear dispersing emulsifier (PRO200, PRO Scientific, USA). A further reduction in size was achieved by treating the mixture for 5 min (using a 5-s on and 5-s off cycle) in an ultrasonic cell pulverizer (JYD-250L, Zhixin Instruments Co., Ltd, China) at 200 W to obtain a nanoemulsion.

### Particle Size and Zeta Potential Analysis

The TET-LCNPs were diluted tenfold with water, and their particle sizes, polydispersity indexes, and zeta potentials were then determined at 25 °C using a Zetasizer (Nano-ZS, Malvern Instruments Ltd., Worcestershire, UK). All determinations were performed in triplicates.

### Determination of Encapsulation Efficiency and Drug Loading

The drug encapsulation efficiency (EE) and drug loading (DL) were determined using ultrafiltration in centrifuge tubes that were fitted with an ultra-filter (Amicon Ultra, MWCO 30 kDa, Millipore Company). About 500 μL of each formulation was placed in the upper chamber, and the tubes were then centrifuged at 5000 rpm for 30 min. The amount of free drug in the filtrate was determined at 283 nm using HPLC (LC20A, Shimadzu, Japan). The mobile phase consisted of methanol/deionized water/triethylamine (90:10:0.7, *v*/*v*/*v*) and was run at a flow rate of 1.0 mL/min. TET was separated using a Dikma C18 column (5 μm, 4.6 × 250 mm). EE and DL were calculated using Eqs. 1 and 2

1 $$ \mathrm{E}\mathrm{E}\%=\frac{W_{\mathrm{total}}-{W}_{\mathrm{free}}}{W_{\mathrm{total}}}\times 100 $$

2 $$ \mathrm{D}\mathrm{L}\%=\frac{W_{\mathrm{total}}-{W}_{\mathrm{free}}}{W_{\mathrm{total}}-{W}_{\mathrm{free}}+{W}_{\mathrm{lipid}}}\times 100, $$

where *W*_total_, *W*_free_, and *W*_lipid_ represent the total amount of drug in the nanoparticles, the amount of drug in the filtrate, and the amount of lipid in the formulation, respectively.

### Morphology

After diluting the samples 100-fold with purified water, one drop of the LCNP suspension was deposited onto a 300-mesh carbon-coated copper grid, and the LCNPs were allowed to settle for 3–5 min. The excess fluid was removed using absorbent paper, and the samples were then visualized using a transmission electron microscope (HT7700, Hitachi Ltd, Tokyo, Japan).

### Small-Angle X-Ray Scattering Measurement

Small-angle X-ray scattering (SAXS) measurements were performed using a Xeuss 2.0 SAXS/WAXS system (Xenocs, France) with pinhole collimation for point focus geometry [18]. The SAXS camera was equipped with a Hi-Star 2D detector. The optics and sample chamber were used under vacuum conditions to minimize air scatter. The liquid samples were placed in 0.5-mm glass capillaries, and measurements were performed in a vacuum at 25 °C with an exposure time of 0.5 h.

### In Vitro Drug Release Test

In vitro drug release was evaluated using the dynamic dialysis bag method [19]. Briefly, 2 mL each of TET solution and the TET-loaded LCNP formulation were loaded separately into dialysis bags. The dialysis bags were then immersed in 70 mL of freshly prepared phosphate-buffered saline (PBS; pH 6.0) at 35 °C. The magnetic stirring speed was 200 rpm. At predetermined time intervals, 2 mL of each sample was withdrawn and immediately replaced with an equal volume of fresh PBS (pH 6.0). The concentration of the drug that had been released by the particles was determined using HPLC.

### In Vitro Corneal Permeation Evaluation

Corneal permeation studies were performed using the corneas that were excised from the rabbits [20]. The rabbits were sacrificed with an injection of air into the marginal ear vein. We then immediately excised the corneas from the rabbits and preserved them in glutathione bicarbonate Ringer’s solution (GBR). An equal volume of each sample (500 μL) was added to the donor chamber, and 4.5 mL of fresh PBS (pH 6.0) was added to the receptor chamber. Samples from the receptor chamber were withdrawn at predetermined time intervals and immediately replaced with an equal volume of fresh PBS (pH 6.0). All analyses were performed as described for the drug encapsulation efficiency evaluations. The experiments were performed in triplicates. The apparent corneal permeability coefficient (*P*_app_) was calculated using Eq. 3 [21] as follows:

3 $$ {P}_{\mathrm{app}}=\frac{\varDelta Q}{\varDelta t\cdot {C}_0\cdot A\cdot 60}, $$

where $\frac{\varDelta Q}{\varDelta t}$ is the slope of the linear portion of the cumulative permeated amount-versus-time plot, *C*_0_ is the initial drug concentration in the donor cell, *A* is the exposed corneal surface area (0.5 cm^2^), and 60 was used to convert minutes to seconds.

### Pre-Ocular Retention Time Study

The pre-ocular retention of the TET-LCNP was assessed using a noninvasive fluorescence imaging system (NightOWL II LB985, Berthold Technologies, Germany) [22]. The LCNP formulation was labeled as follows: TET was replaced with rhodamine B (Rh B) in the oil phase, and the formulation was then processed using the same method that was used to prepare the LCNPs. The albino rabbits were examined while conscious. Immediately before imaging, the animals were anesthetized using chloral hydrate (injection 2.5 mL/kg) via the ear vein. Precisely 20 μL of the formulation was injected directly into the conjunctival sac of the right eye, and the left eye was used as the control (Rh B solution). The eyes were manually closed for 10 s to allow the solution to be distributed over the cornea. After 0, 0.17, 0.5, 1.0, 1.5, 2.0, and 2.5 h, imaging was performed. The region of interest (ROI) for the residual fluorescence was around the ocular and non-ocular areas. The remaining intensity (*R*) was calculated according to Formula 4 as follows:

4 $$ R\%=\frac{A-B}{C}\times 100, $$

where *A* was the intensity of ROI, *B* was the background fluorescence intensity, and *C* was the intensity of the ROI at 0 h [22].

### Pharmacokinetic Study in Aqueous Humor

A pharmacokinetic study was performed on aqueous humor fluids using microdialysis [23]. Briefly, three rabbits were locally anesthetized via an injection of 2.5 mL/kg of chloral hydrate into the marginal ear vein. A microdialysis probe (MD 2000, Bioanalytical Systems, Inc., West Lafayette, IN) was implanted into the anterior chamber of the eye using a 20-G needle. The needle was then removed, leaving the probe with the microdialysis membrane in the middle of the anterior chamber. Thereafter, the probe was perfused with PBS (pH 6.0) at a flow rate of 3 μL/min using a microdialysis pump (CMA106, CMA Microdialysis Co., Ltd., Stockholm, Sweden). The corneal wound surfaces were treated with 0.3 % (*w*/*v*) ofloxacin ophthalmic drops and allowed to stabilize for 24 h before any agent was administered.

The dialysate was collected at 15 min after the 30 min perfusion was completed. In vivo recovery (*R*) was calculated using Eq. 5

5 $$ R\%=\frac{C_{\mathrm{d}}-{C}_{\mathrm{p}}}{C_{\mathrm{m}}-{C}_{\mathrm{p}}}\times 100, $$

where *C*_d_, *C*_p_, and *C*_m_ are the concentrations of the drug in the dialysate, perfusate, and aqueous humor, respectively. *R* is the value of the slope of the plot of (*C*_d_ − *C*_p_) versus *C*_p_.

Next, 0.2 mL of each formulation (1.0 mg/mL) was injected into the eyes of separate groups of rabbits. Dialysates were collected every 20 min for the first 2 h and every 30 min thereafter after instillation. Each experiment was repeated three times, and all samples were analyzed using HPLC.

### Statistical Analysis

The optimized TET-LCNPs and all data were analyzed using Design-Expert software (8.06 version, Stat Ease, Inc., Minneapolis, MN) [24]. The results are presented as the mean ± standard deviation. Statistical analyses of the results were performed using two-way analysis of variance and one-way ANOVA in *SPSS* software (version 17.0, SPSS Inc., IL, USA). Statistical significance was established at *p* < 0.05 [25].

## Results and Discussion

### Central Composite Factorial Design

A quadratic polynomial equation was used to determine the responses according to differences in the independent variables, as shown in Eq. 6

6 $$ Y={A}_0+{A}_1{X}_1+{A}_2{X}_2+{A}_3{X}_3+{A}_4{X}_1{X}_2+{A}_5{X}_2{X}_3+{A}_6{X}_1{X}_3+{A}_7{X_1}^2+{A}_8{X_2}^2+{A}_9{X_3}^2, $$

where *Y* is the dependent variable, *X*_1_–*X*_3_ are the independent variables, and *A*_1_–*A*_9_ are the regression coefficients of the respective variables.

The best-fit model for the responses was the quadratic model, which had the maximal *r*-value. The correlation coefficient (*r*) was used as quality indicator to evaluate the fitness of the second-order polynomial equation. ANOVA was performed to evaluate the impact of the quadratic model terms on response and quantitative effects, as shown in Table 2. The respective *p* values shown in Table 2 indicate that the amount of TET (*X*_1_) and the amount of GMO (*X*_2_) had significant effects (*p* < 0.05) on responses but that the effect of the F127/GMO ratio (*X*_3_) was not significant for EE and DL.

**Table 2:** Regression coefficients of the quadratic equations in optimization experiment

Parameters	Regression coefficient			
	EE (%)	*p*	DL (%)	*p*
*A*_0_	80.90		0.94	
*A*_1_	3.33	<0.001	0.27	<0.001
*A*_2_	1.48	0.026	−0.09	<0.001
*A*_3_	−0.54	0.365	−1.07 × 10^−3^	0.953
*A*_4_	0.22	0.770	5.00 × 10^−3^	0.831
*A*_5_	0.22	0.899	−2.50 × 10^−3^	0.299
*A*_6_	−0.10	0.775	−0.03	0.915
*A*_7_	1.70	0.012	0.06	0.009
*A*_8_	−1.46	0.024	−6.59 × 10^−3^	0.706
*A*_9_	−0.61	0.293	0.01	0.529
*r*	92.85		98.26	
*p*	0.0028		<0.0001	

*A*_0_–*A*_9_ are the regression coefficients of the respective variables. The *r*-value was used as a quality indicator to evaluate the fitness of the second-order polynomial equation. The *p* value less than 0.05 was considered to be statistically significant

**Fig. 1:**
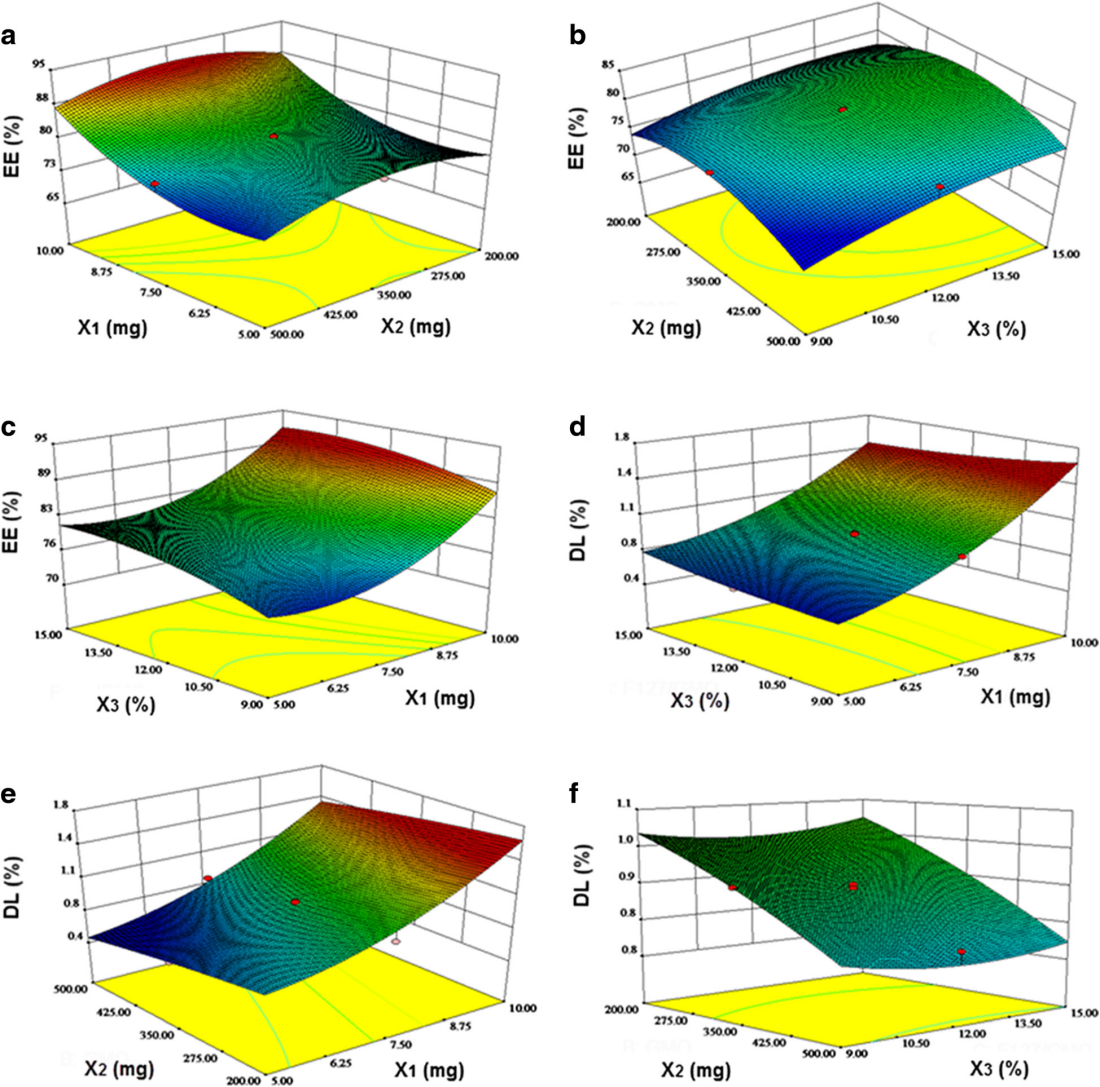
RSM demonstrating the effect of the independent variables on EE and DL. *X*_1_ represents the amount of TET, *X*_2_ represents the amount of GMO, and *X*_3_ represents the ratio between F127 and GMO. **a** Central composite design-response surface for the effect of the amount of TET (*X*_1_) and the amount of GMO (*X*_2_) upon EE. **b** Central composite design-response surface for the effect of the amount of GMO (*X*_2_) and the F127/GMO ratio (*X*_3_) upon EE. **c** Central composite design-response surface for the effect of the amount of TET (*X*_1_) and the F127/GMO ratio (*X*_3_) upon EE. **d** Central composite design-response surface for the effect of the amount of TET (*X*_1_) and the F127/GMO ratio (*X*_3_) upon DL. **e** Central composite design-response surface for the effect of the amount of TET (*X*_1_) and the amount of GMO (*X*_2_) upon DL. **f** Central composite design-response surface for the effect of the amount of GMO (*X*_2_) and the F127/GMO ratio (*X*_3_) upon DL

The sign and value of the quantitative effect represented the tendencies and the magnitudes of the responses, respectively. EE and DL were the inputs used to construct the respective response surface models (RSMs). Response surface analyses were also plotted in three-dimensional model graphs. The three-dimensional response surface plots for EE and DL are presented in Fig. 1. By analyzing these graphs, the optimal parameters for the TET-LCNP formulation were found to be 10 mg TET, 308.99 mg GMO with F127-to-GMO ratio of 12.99:100 (*w*/*w*), 0.1 % (*w*/*v*) Gelucire 44/14, 30 mg QACMC, and 30 mL water. Table 3 shows the comparison between the actual and predicted data obtained from the quadratic models. The proximity of the actual and predicted values demonstrated the high validity and adequacy of the model. This formulation was considered to be the most appropriate formulation for ophthalmic application, and it was therefore used for further characterizations.

**Table 3:** Predicted values and actual values of optimal prescription of TET-LCNPs

Parameter	Predicted values (%)	Actual values (%)	Deviation (%)
EE	91.55	95.46	−4.27
DL	1.57	1.63	−3.82

Deviation (%) = (predicted values − actual values)/predicted values × 100 %

A central composite factorial design is a well-established approach that is used in pharmaceutical formulation development and optimization because it allows the maximal amount of information to be extracted from a small number of well-designed experiments. The central composite design technique is a very useful tool that provides statistical models, which help researchers to understand the interactions between the parameters that have been optimized and also helps them to optimize the effective parameters using the minimum number of experiments [26, 27]. This approach is therefore more efficient and economical than using other designs [28].

### Particle Size and Zeta Potential

**Fig. 2:**
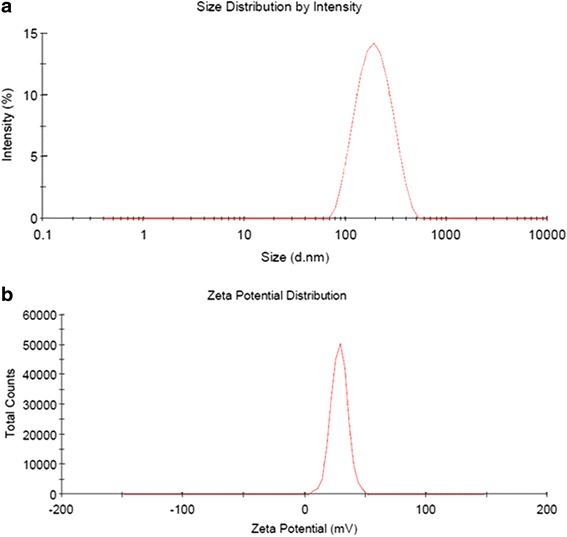
Characteristics of TET-LCNPs. **a** Particle size distribution. **b** Zeta potential distribution. The TET-LCNPs were diluted tenfold with water; particle size distribution and zeta potential were determined at 25 °C. All determinations were performed in triplicates

Nanoparticle size has an important effect on the dispersion of ophthalmic formulations. The mean diameter of the TET-LCNPs was 170.0 ± 13.34 nm, the mean polydispersity index was 0.166 ± 0.02 (Fig. 2a), and the distribution of particle sizes was relatively narrow. These properties are advantageous for the uptake of TET-LCNPs through the cornea. Many studies have attempted to determine the optimal particle size for ophthalmic formulations, and a narrow size range ensures a lower level of irritation, adequate bioavailability, and good compatibility with ocular tissues [29]. Sonication is a powerful approach that can be used to decrease particle size, but one disadvantage of this technique is that strong mechanical treatment can also degrade the lipid-water structure and reduce drug loading capacity. The optimal formulation had a positive surface charge of 29.3 ± 1.25 mV (Fig. 2b). When the cationic material QACMC was added, the zeta potential of the system was sufficiently high to provide effective electric repulsion to avoid the aggregation of particles [30]. Furthermore, F127 was used as a stabilizer, and it also had a desirable effect in that it increased TET-LCNPs stability [31].

### Encapsulation Efficiency and Drug Loading

The EE was 95.46 ± 4.13 %, and the nanoparticles showed 1.63 ± 0.07 % DL, indicating that most of the drug was encapsulated in the liquid crystalline nanoparticles.

### Morphology of TET-LCNPs

**Fig. 3:**
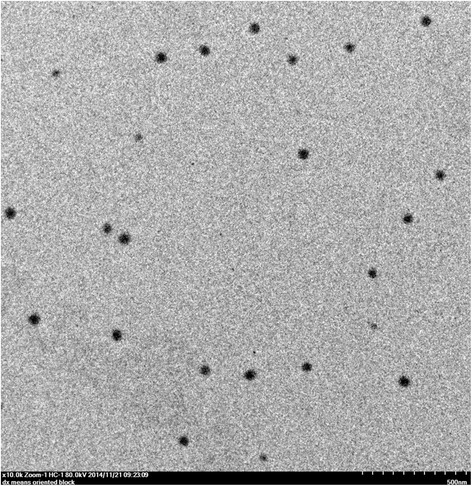
Photograph of TET-LCNPs taken using transmission electron micrograph. After diluting the samples 100-fold with purified water, one drop of the LCNP suspension was deposited onto a 300-mesh carbon-coated copper grid, and the LCNPs were allowed to settle for 3–5 min. The excess fluid was removed using absorbent paper, and the samples were then visualized using a transmission electron microscope

When the samples were visualized using a transmission electron microscope, spherically shaped particles with smooth surfaces were observed, as shown in Fig. 3. These findings indicated the successful formation of TET-LCNPs.

### SAXS Measurement

**Fig. 4:**
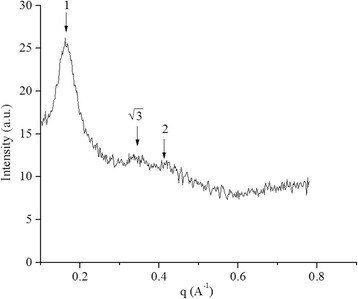
SAXS profiles of the TET-LCNPs. The SAXS camera was equipped with a Hi-Star 2D detector. The optics and sample chamber were used under vacuum conditions to minimize air scatter. The liquid samples were placed in 0.5-mm glass capillaries, and measurements were performed in a vacuum at 25 °C with an exposure time of 0.5 h

SAXS was performed to determine the structural organization of the LCNP formulations. Several Bragg peaks are shown in Fig. 4. The spacing ratio of the reflections was 1: $\sqrt{3}$: 2 [32], which indicates the inverted hexagonal (H_2_) phase. Many studies have explored using the dispersed L_2_ phase and V_2_ phase in ocular drug carriers [33]. However, only a small amount of evidence has support the notion that the H_2_ phase can be a valuable carrier for an ocular delivery system. In this study, in the H_2_ phase, the amphiphilic molecules were organized as nearly cylindrical aqueous tubes. This study has indicated that the structure of the H_2_ phase, in terms of its high internal interfacial area, is separated into hydrophilic and hydrophobic domains. This finding suggests a potential method for encapsulating hydrophilic, lipophilic, and amphiphilic drugs [34]. Because it possesses these properties, the H_2_ phase may be a promising candidate for TET delivery systems.

### In Vitro Release of TET-LCNPs

**Fig. 5:**
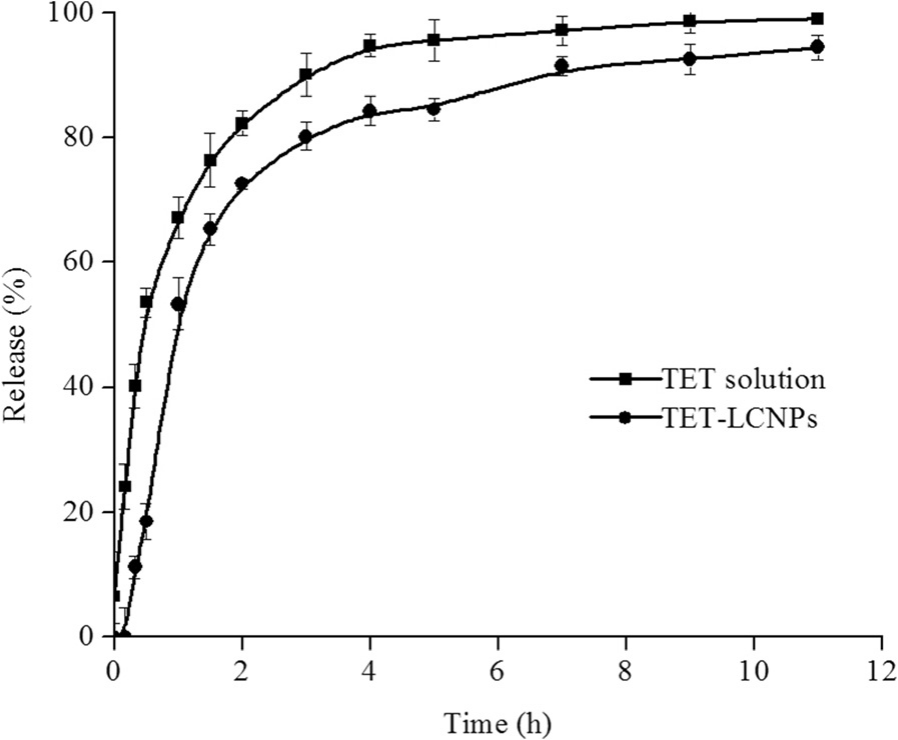
In vitro release profiles of TET released from a TET solution and from TET-LCNPs (*n* = 3). In vitro drug release was evaluated using the dynamic dialysis bag method. Two milliliters of each of TET solution and the TET-loaded LCNP formulation was loaded separately into the dialysis bags. The dialysis bags were then immersed in 70 mL of freshly prepared phosphate-buffered saline (PBS, pH 6.0) at 35 °C. The magnetic stirring speed was 200 rpm

The in vitro release profiles for the release of TET from the TET solution and from the TET-LCNPs are illustrated in Fig. 5. In this study, PBS (pH 6.0) was selected to enhance the solubility of TET. TET rapidly diffused from the TET solution, with approximately 53.60 % of the drug being released within 0.5 h. However, in the LCNP formulations, only 18.50 % of the drug was released within the first 0.5 h, during which the LCNPs exhibited an initial burst release of the drug. It is clear that after the initial burst release from the LCNPs, which lasted approximately 1.5 h, the rate of TET release from the LCNPs slowed and was close to the Ritger-Peppas release (*r* = 0.9580). After 12 h, approximately 94.40 % of the TET had been released from the LCNPs, while 99.00 % of the TET had been released from the TET solution. The initial burst in the release of the drug may have resulted from the release of free TET that had adsorbed onto the nanoparticle surface. Adsorbed TET would easily diffuse from the matrix, while TET that had been incorporated into the nanoparticle core would be released over a prolonged period [35]. Both the initial burst release and the sustained-release portions of the drug release profile are of interest for clinical applications. The initial burst is useful for improving drug permeation and for pushing for a faster time of onset for drug activity, while the sustained release of the drug allows it to be administered over a more prolonged period of time.

### In Vitro Corneal Penetration Evaluation

A corneal penetration experiment was performed to evaluate the ability of TET to penetrate across the cornea when administered in either a TET solution or in a solution of TET-LCNPs containing the same concentration TET. LCNPs considerably increased the ability of TET to penetrate the cornea. The *P*_app_ values of the TET solution and the TET-LCNPs were 1.23 × 10^−5^ and 2.50 × 10^−5^ cm/s, respectively. The *P*_app_ was 2.03-fold higher for the TET-LCNPs than for the TET solution, suggesting that more TET was taken up by the rabbit corneas when TET-LCNPs were applied than when the TET solution was applied. One potential reason that LCNPs might enhance corneal permeation is the bioadhesive property of the liquid crystalline nanoparticles. Their extremely small particle size and increased surface area may promote the permeation of the drug. Another potential reason is that the H_2_ phase, a promising candidate for drug delivery systems, may permit the incorporation of more drugs independent of drug solubility and thereby improving drug permeation [36]. Moreover, Gelucire 44/14 has been reported to be an effective enhancer of corneal permeation [37]. Thus, combining LCNPs and Gelucire 44/14 might result in significantly improved corneal penetration.

### Pre-Ocular Retention of LCNPs

**Fig. 6:**
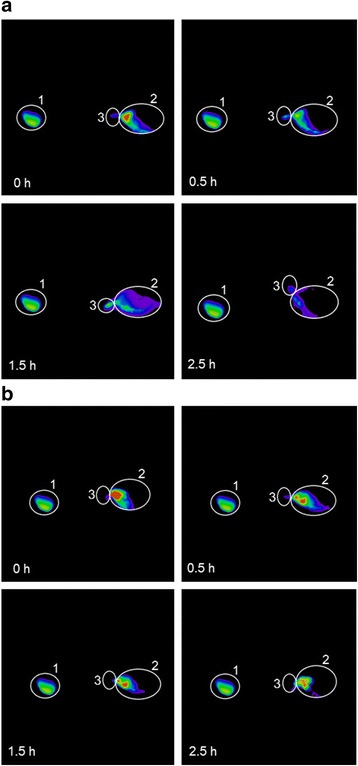
Fluorescence images of rabbit eyes after administration of the formulations. **a** Rh B solution and (**b**) Rh B-LCNPs. *1* ocular ROI, *2* intensity reference standard, *3* inner canthus and nasolacrimal duct region

As shown in Fig. 6a, rapid drainage was observed in the rhodamine B (Rh B) solution group following the administration of the drug. After 1.5 h, nearly no fluorescence was observed in the ROI, and only a small amount of fluorescence intensity remained in the lachrymal duct. However, when the eyes were instilled with Rh B-LCNPs, the diffusion of the drug was not as rapid in the pre-corneal region, and relatively strong fluorescence intensity was observed in the ROI for up to 1.5 h, as shown in Fig. 6b.

**Fig. 7:**
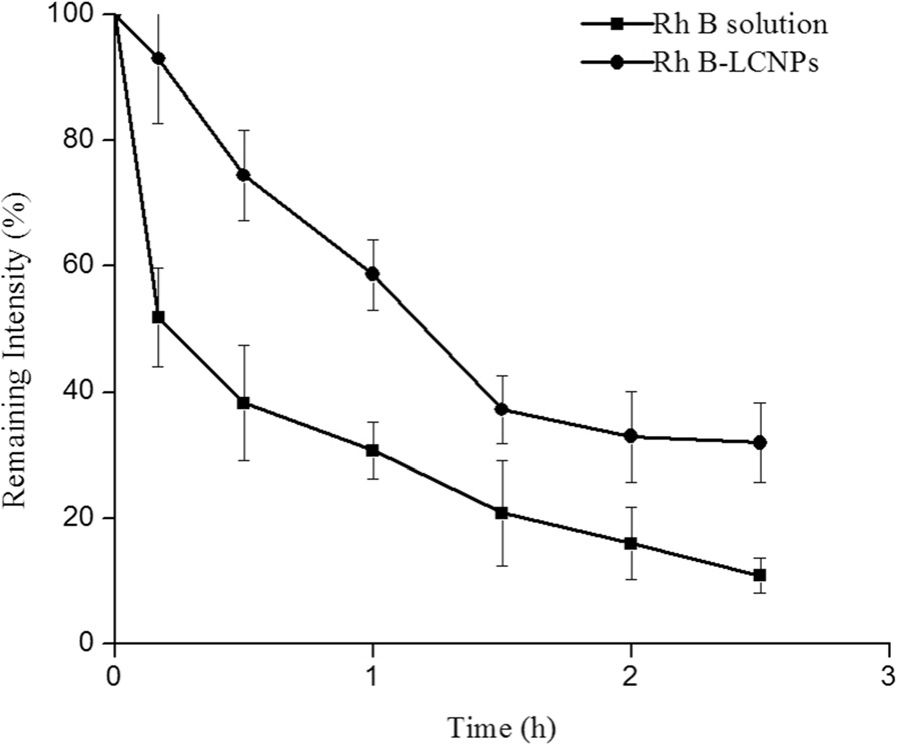
Pre-ocular retention of various formulations (*n* = 3). Remaining intensity (*R*) was calculated according to the equation: *R* = (*A* − *B*)/*C* × 100 %, where *A* was the intensity of ROI, *B* was the background fluorescence intensity, and *C* was the intensity of ROI at 0 min

Fluorescence intensity remaining in the ROI at 0.5 h after administration was defined as *A*_0.5_. At 0.5 h, only 38.31 % fluorescence intensity remained in the ROI, which was significantly lower than that of Rh B-LCNPs, *A*_0.5_ of Rh B-LCNPs was 74.40 %, as displayed in Fig. 7. At 1.5 h, the statistical analysis noted that 20.79 and 37.19 % intensity remained in the Rh B solution and Rh B-LCNPs group, respectively. A summary of statistical analysis is shown in Table 4. The area under the concentration-time curve (AUC) of the LCNP formulation was significantly higher than that of solution (*p* < 0.01). There was about a 1.83-fold increase in AUC of Rh B-LCNPs compared to that of Rh B solution. The clearance of Rh B-LCNPs in the initial phase was much slower, as elimination rate constant (*K*_e_) was significantly lower than that of the Rh B solution (*p* < 0.05). The *K*_e_ value for Rh B solution was 1.30-fold more than that obtained for the Rh B-LCNPs.

**Table 4:** Pre-corneal clearance parameters ($\overline{X}\pm \mathrm{S}\mathrm{D},$ *n* = 3)

Preparation	AUC (% h)	*K*_e_ (h^−1^)
Rh B solution	73.83 ± 10.32	0.65 ± 0.06
Rh B-LCNPs	135.01 ± 17.07**	0.50 ± 0.01*

*AUC* area under the curve, *K*_e_ elimination rate constant

^*^ *p* < 0.05 versus Rh B solution; ^**^ *p* < 0.01 versus Rh B solution

These results indicate that the application of the LCNP formulation resulted in relatively strong fluorescence intensity and slow clearance from the ROI. Adding the cationic material QACMC increased the viscosity of the aqueous phase, which hindered the rapid dispersion of the particles from the corneal surface [38]. The addition of positively charged QACMC resulted in electrostatic adhesion or interactions with negatively charged mucin in the corneal epithelium, which increased the ocular residence time of the drug on the corneal surface [39]. Rh B as a fluorescent dye was encapsulated in the nanoparticles, which can reflect retention capacity of LCNPs [22]. Rh B has many advantages as a fluorescent dye, such as cheap and easy to get, good light stability, and high fluorescence quantum yield [40].

### Pharmacokinetic in Aqueous Humor

In vivo probe recovery was evaluated before the pharmacokinetic study to ensure that the implanted probes were sufficiently functional. The in vivo recovery rate was 46.08 ± 1.93 % (*n* = 3).

**Fig. 8:**
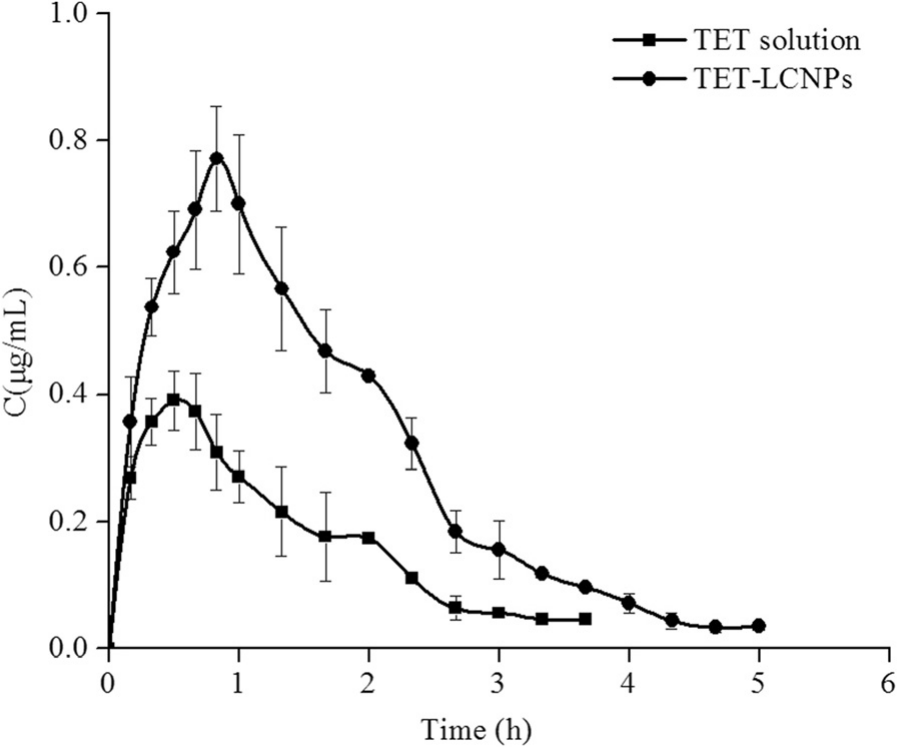
TET concentration-time profiles when 200 μL was topically administered at a dose of 1.0 mg/mL in the aqueous humor (*n* = 3)

The aqueous humor pharmacokinetics parameters are summarized in Table 5. As shown in Fig. 8, the AUC value was 2.65-fold higher for the TET-LCNPs than for the TET solution (*p* < 0.01), and the peak concentration (*C*_max_) for the TET-LCNPs was 2.08-fold higher (*p* < 0.01). The time to peak concentration (*T*_max_) and the half-time (*t*_1/2_) of the TET-LCNPs were both longer than the same values for the TET solution. The *t*_1/2_, *C*_max_, and AUC values for the TET-LCNPs were significantly different from the corresponding values for the TET solution (*p* < 0.01).

**Table 5:** Pharmacokinetic parameters of TET in aqueous humor after topical administration in the conscious rabbits ($\overline{X}\pm \mathrm{S}\mathrm{D},$ *n* = 3)

Preparation	*T*_max_ (h)	*C*_max_(μg mL^−1^)	AUC (μg mL^−1^ h)	*t*_1/2_ (h)
TET solution	0.50 ± 0.10	0.41 ± 0.03	0.61 ± 0.03	0.85 ± 0.01
TET-LCNPs	0.83 ± 0.10*	0.86 ± 0.03**	1.64 ± 0.07**	1.41 ± 0.10**

*T*_*max*_ time to peak concentration, *C*_*max*_ maximal concentration of TET, *AUC* area under the curve, *t*_*1/2*_ half-time

^*^ *p* < 0.05 versus TET solution; ^**^ *p* < 0.01 versus TET solution

The enhanced ocular bioavailability that was provided by the LCNP delivery system may result from three factors: the ocular contact time of the delivery system, the ability of the drug to permeate through the cornea, and a higher drug loading capacity. First, the pre-ocular retention study indicated that the LCNP formulation resulted in prolonged residence in the ROI, which may have lengthened the ocular contact time. Second, our in vitro study of corneal penetration showed that the *P*_app_ of the LCNP formulations containing TET was 2.03-fold higher than the *P*_app_ for the TET solution. Third, our results show that the H_2_ phase is a promising candidate because it possesses specific structural properties, including its high packing density and its extremely long and straight water-filled rods, which can accommodate a greater amount of the drug, resulting in improved drug permeability through the cornea [41]. Furthermore, Gelucire 44/14 enhances the absorption of poorly soluble drugs, which contributes to improved drug bioavailability. Together, these factors ensure intimate contact between the drug and the epithelial mucosal surface of the eye and prevent tear washout, and they are consequently associated with a sustained drug release profile and prolonged drug retention times. Using this drug delivery system might be more advantageous than using conventional dosage forms of TET because it is associated with increased solubility and bioavailability. It might also improve patient compliance because of its sustained effects and reduce the required dose, consequently reducing any associated side effects.

## Conclusions

In summary, the present study demonstrates the potential of a TET-LCNP drug delivery system to increase drug solubility and improve bioavailability. The optimal formulation of TET-LCNPs was found to be nanosized and uniformly dispersed. The in vitro results showed that TET-LCNPs were associated with a more sustained release profile than those achieved using a TET solution. The corneal penetration study revealed that LCNPs significantly enhanced the transcorneal permeation of TET. The enhancement of transcorneal permeation was further supported by the results of an in vivo pharmacokinetics study. The LCNP system is therefore a potential approach for improving the ocular bioavailability of TET by enhancing its ocular retention and corneal permeation.

### Ethics Statement

New Zealand white rabbits (2.5–3.0 kg) were provided by the Chinese Academy of Medical Sciences of Radiation Research Institute (License No.: SCXK JIN 2013-0007). The animals, housed in standard cages in a light-controlled room at 25 ± 1 °C and 50 ± 5 % relative humidity, were fed with a standard pellet diet and water ad libitum. Procedures involving animal care and management were reviewed and approved by the Animal Ethical Committee at Tianjin University of Traditional Chinese Medicine (document number TCM-LAEC 2014007).

## Abbreviations

AUC: the area under the concentration-time curve
*C*_max_: concentration at peak
DL: drug loading
EE: entrapment efficiency
F127: poloxamer 407
GBR: glutathione bicarbonate Ringer’s solution
GMO: glyceryl monooleate
*K*_e_: elimination rate constant
LCNPs: liquid crystalline nanoparticle system
*P*_app_: the apparent corneal permeability coefficient
QACMC: amphipathic octadecyl-quaternized carboxymethyl chitosan
R: recovery
Rh B: rhodamine B
ROI: the region of interest
RSM: response surface model
SAXS: small-angle X-ray scattering
*t*_1/2_: half-time
TET: tetrandrine
TET-LCNPs: TET-loaded LCNPs
*T*_max_: the time to peak concentration
